# Comprehensive Bioinformatics Analysis Combined with Wet-Lab Experiments to Find Target Proteins of Chinese Medicine Monomer

**DOI:** 10.3390/molecules27186105

**Published:** 2022-09-19

**Authors:** Xiaohui Xu, Yunyi Zhu, Changling Yue, Qianwen Yang, Zhaohuan Zhang

**Affiliations:** 1 School of Preclinical Medicine, Wannan Medical College, Wuhu 241002, China; 2 School of Life Sciences, Shanghai University, Shanghai 200444, China; 3 Department of Laboratory Medicine, Changzheng Hospital, Naval Medical University, Shanghai 200003, China

**Keywords:** bioinformatics, transcription factors, reverse docking, daphnoretin, JAK2 agonist, JAK2/STAT3, Chinese medicine monomer, NSCs, OPCs, multiple sclerosis

## Abstract

How to use bioinformatics methods to quickly and accurately locate the effective targets of traditional Chinese medicine monomer (TCM) is still an urgent problem needing to be solved. Here, we used high-throughput sequencing to identify the genes that were up-regulated after cells were treated with TCM monomers and used bioinformatics methods to analyze which transcription factors activated these genes. Then, the binding proteins of these transcription factors were analyzed and cross-analyzed with the docking proteins predicted by small molecule reverse docking software to quickly and accurately determine the monomer’s targets. Followeding this method, we predicted that the TCM monomer Daphnoretin (DT) directly binds to JAK2 with a binding energy of −5.43 kcal/mol, and activates the JAK2/STAT3 signaling transduction pathway. Subsequent Western blotting and in vitro binding and kinase experiments further validated our bioinformatics predictions. Our method provides a new approach for quickly and accurately locating the effective targets of TCM monomers, and we also have discovered for the first time that TCM monomer DT is an agonist of JAK2.

## 1. Introduction

Multiple sclerosis is an autoimmune disease characterized by inflammatory demyelination of the central nervous system. It is a common non-invasive and crippling nervous system disease in young adults all over the world [1]. There are more than 2.5 million patients worldwide [2]. For a long time, the treatment of MS has been faced with great challenges. At present, there is no therapy directly targeting promoting remyelination, though there are some drugs targeting immune modulation to reduce myelin attack [1]. Oligodendrocyte precursor cells (OPCs) are the cell source for regeneration after demyelination. After demyelination, OPCs are activated, proliferate and are involved in partial remyelination in the early stage of MS. With the progress of the disease, remyelination eventually fails [3], though at present the mechanism of failure of remyelination in chronic MS is not fully understood. However, it is believed that the senescence of endogenous OPCs plays an important role [4,5]. The other cell origin for remyelination is endogenous neural stem cells (NSCs). It has been shown that NSC-mediated oligodendrogenesis in response to growth factors declines with age [6], and the NSCs’ cellular senescence is responsible for diminished remyelination potential in progressive multiple sclerosis (MS) patients [4].

Therefore, an adequate supply of exogenous and young OPCs or NSCs may be beneficial for the remyelination in MS patients.

It has been found that NSCs can produce oligodendrocytes (OLs) to participate in the regeneration and repair of the myelin sheath after transplant. However, in the absence of exogenous intervention, the production efficiency of myelinated OLs forming NSCs is low, tissue reconstruction and repair are very slow or stagnant, and it is difficult to effectively reverse the progress of MS patients after NSCs transplant [7].

Therefore, in vitro induction of NSCs to differentiate into OPCs, followed by transplant of the induced OPCs to MS patients, may be a reasonable method to treat the disease. Overexpression of oligodendrocyte-specific transcription factors, such as Ascl1, Olig2, and Zfp488 in NSCs, have been reported to promote NSC differentiation into oligodendrocytes [8,9,10]. Cocktails of various growth factors have been used to produce homogenous populations of OPCs from NSCs in vitro [11]. However, these have disadvantages such as the risk of tumor formation caused by the introduction of foreign genes, and the complexity of conditions that induce the production of OPCs by cytokine combinations. Clearly, safer, more efficient and selective approaches are needed to direct differentiation of NSCs to OPCs.

In the present study, we found a small molecule, daphnoretin (DT), from a TCM library, that could significantly promote cultured NSCs to differentiate to OPCs. Subsequently, we used comprehensive bioinformatics analysis combined with wet-lab experiments and found that DT promoted the directional differentiation of NSCs to OPCs by directly binding to JAK2 and activating the JAK2/STAT3 signal transduction pathway.

## 2. Results

### 2.1. DT Promotes the Generation of OPCs from NSCs

**Figure 1:**
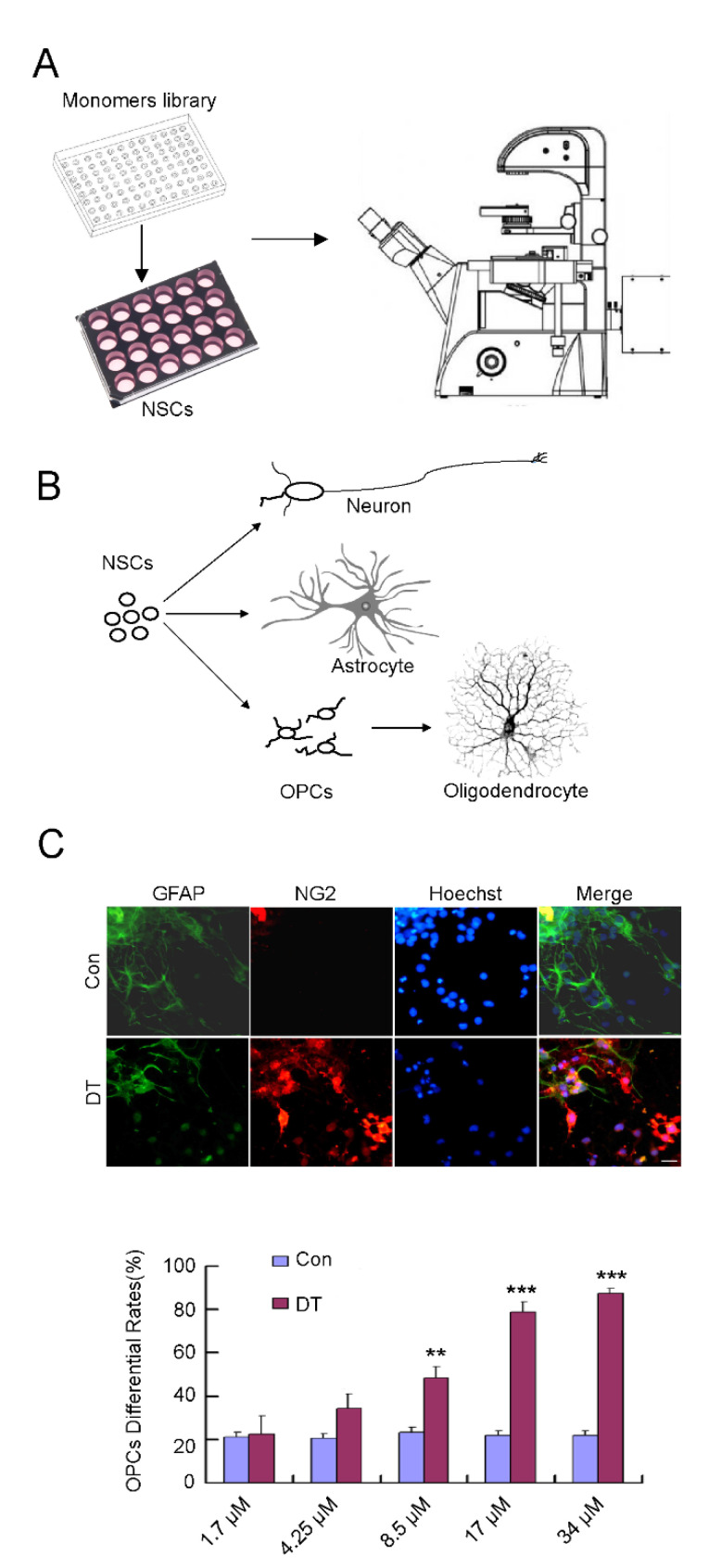
(**A**) DT promotes the generation of OPCs from NSCs. Scheme of Chinese herb monomer library screening in NSC culture. Scheme of NSCs differentiation. (**B**) NSCs differentiate into three types of cells with distinct morphology: neurons, astrocytes and OLs. (**C**) NSCs treated with or without DT had different OPC differentiation rate. Cells were immunostained for NG2 (red) or GFAP (green), and Hoechst (blue) was used to label cell nuclei. DT treatment dramatically increased the generation of NG2-positive cells. The ratio of NG2-positive cells derived from NSCs was determined after cultures were treated with DT at different concentrations. **, *p* < 0.01, ***, *p* < 0.001 by Student’s *t*-test.

NSCs are generally considered as tri-potent, self-renewing progenitors that can generate neurons, astrocytes, and oligodendrocytes (OLs), the three major cell types of the CNS (Figure 1B). They have been used in transplant treatments for CNS disease [12,13]. To identify compounds that can induce the selective differentiation of NSCs, we carried out a screen based on a compound library purified from traditional Chinese herbs (Table S1). To carry out the primary screen, NSCs were treated with 20 μM concentrations of compounds after plating in neurobasal with 2% B27. After 10 days, cultures were examined under a phase-contrast microscope (Figure 1A). We found that DT could markedly enhance the generation of round bi-polar and multi-polar morphology cells (Figure 1B,C). To further confirm the effect of DT, neurospheres were plated on a cell culture dish and cultured for 10 days with 20 μM DT. We next performed immunostaining of these cells with NG2 (a specific marker for OPCs) and GFAP(a specific marker for astrocytes) (Figure 1C), and the results showed the DT treatment dramatically increased the generation of NG2-positive cells. NG2 positive cells were 21.91% of the total cells treated with DMSO, but were 22.48, 34.46, 48.35, 78.67, and 87.56% when treated with DT in concentrations of 1.7, 4.25, 8.5, 17, and 34 μM, respectively (Figure 1C). We also detected the expression of O4 (a premature-oligodendrocyte marker) and RIP (a mature oligodendrocyte marker). Most of these cells were characterized as O4 positive, but not RIP-positive (Figure S1A,B), indicating that DT induces the generation of infantile OLs.

### 2.2. Transcriptome Analysis in DT-Induced NSCs Differentiation to OPCs

**Figure 2:**
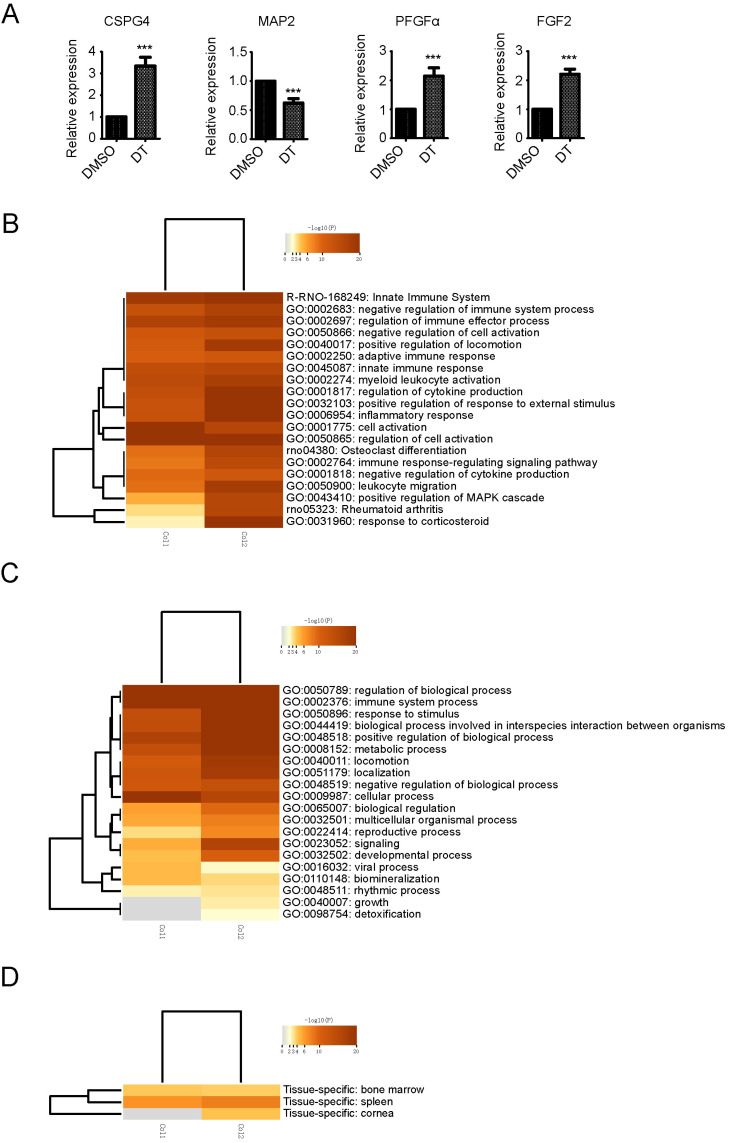
Transcriptome analysis of NSCs at day 3 of DT treatment and gene ontology terms enriched in up-regulated genes. Fold changes of the respective genes in NSCs treated with DMSO or DT. Comparing the expression obtained from mRNA-Seq, values of the reads were normalized with the average values of the housekeeping genes, ***, *p* < 0.001 by Student’s *t*-test (**A**). Heatmap of enriched terms across the top-level gene Ontology molecular function (**B**), biological processes (**C**) and cell type (**D**) among DMSO-NSCs and DT-NSCs, colored by *p*-values.

To investigate the mechanism underlying DT-induced differentiation of OPCs from NSCs, whole transcriptome sequencing analysis was performed. We used a two-fold change as a cut-off line to consider the differential expression of a gene as significant (Tables S2 and S3). The gene CSPG4, which encodes NG2 was remarkably up-regulated, the neuron specific marker gene was significantly down-regulated, and FGF2 and PDGFα which promote OPCs production were also significantly up-regulated (Figure 2A). The above results confirmed the immunostaining results that DT promotes the generation of OPCs from NSCs.

To further analyze the mechanism of gene expression changes involved in the process of DT promoting the differentiation of NSCs into OPCs, all the differentially regulated genes were selected for gene ontology (GO) enrichment annotation and were analyzed in Metascope [14]. Non-redundant GO terms (after semantics similarity) that were significantly over-represented in the list of up-regulated genes in DT treated NSCs were obtained. Figure 2B lists the main GO terms that were enriched in the differentially regulated genes. The main non-redundant GO terms enriched in the DT treated group were related to inflammatory response, cytokine production, immune effector process, cell migration, rheumatoid arthritis, and spinal cord injury (Figure 2B). Differentiation of NSCs into OPCs may involve many changes in other molecular processes. Consequently, other GO terms were also annotated during differentiation (Figure 2C). The results of tissue-specific gene expression enrichment analysis showed that the up-regulated changes of gene expression after DT treatment of NSCs were similar to those of bone marrow cells, because bone marrow is the main location of related blood inflammatory cells, which further indicated that the gene expression promoted by DT treatment of NSCs was mainly associated with inflammatory signaling (Figure 2D). We then focused on the GO terms related to the inflammatory response, cytokine production, the immune effector process, and rheumatoid arthritis, as these processes are crucial for NSC differentiation into OPCs. The relevant GO terms that were enriched, such as cytoskeleton organization, metabolic process, growth and cell migration, were related to the undifferentiated growth and cell movement.

### 2.3. Transcription Factor Analysis

It is generally believed that small compound molecules promote or inhibit the activity of proteins by directly binding to proteins, and finally promote the expression of differential genes through changes in the activity of transcription factors, thereby changing cell fate.

**Figure 3:**
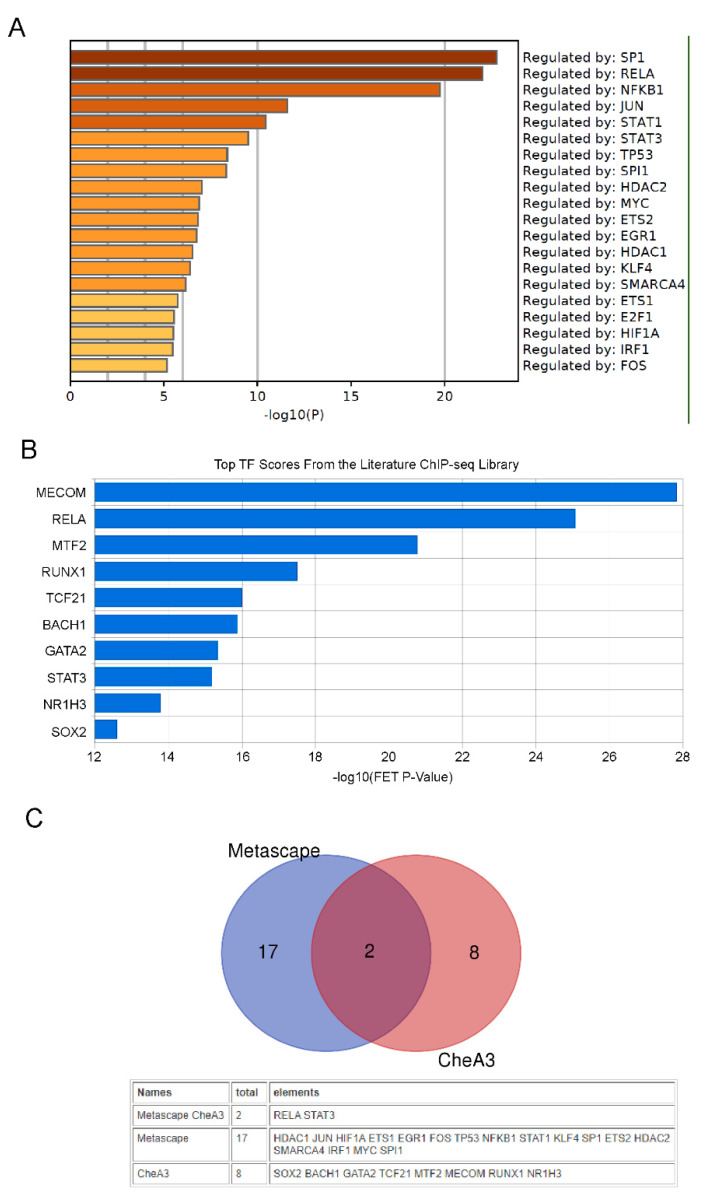
Prediction of transcription factors (TFs) responsible for observed changes in gene expression by Metascape and CheA3 TF enrichment analysis tools. DT treated NSCs up-regulated genes governed by TFs predict by Metascape (**A**). DT treated NSCs up-regulated genes governed by TFs predict by CheA3 (**B**). Venn diagram showing TF overlap (RELA and STAT3) between Metascape and CheA3 (**C**).

Specific transcription factors have crucial roles in NSC differentiation. To gain insight into the specific transcription factors that may be associated with the observed gene expression changes, and potentially play roles in the OPCs differentiation, the differentially expressed genes were subjected to transcription factor enrichment analysis using Metascape and ChEA3 [14,15]. Figure 3A,B shows the top ranked enriched transcription factors from Metascape or ChEA3 analysis with significantly activated TFs in DT treated NSCs. By taking the intersection of the set of activated transcription factors predicted by these two methods, we believe that RELA and STAT3 are the transcription factors most likely to be activated by DT (Figure 3C). Consistent with the results of the above-mentioned gene expression enrichment analysis, the activation of RELA and STAT3 mainly promotes the expression of genes related to inflammatory signaling pathways.

### 2.4. Bioinformatics Predicts That DT Is a JAK2 Agonist

**Figure 4:**
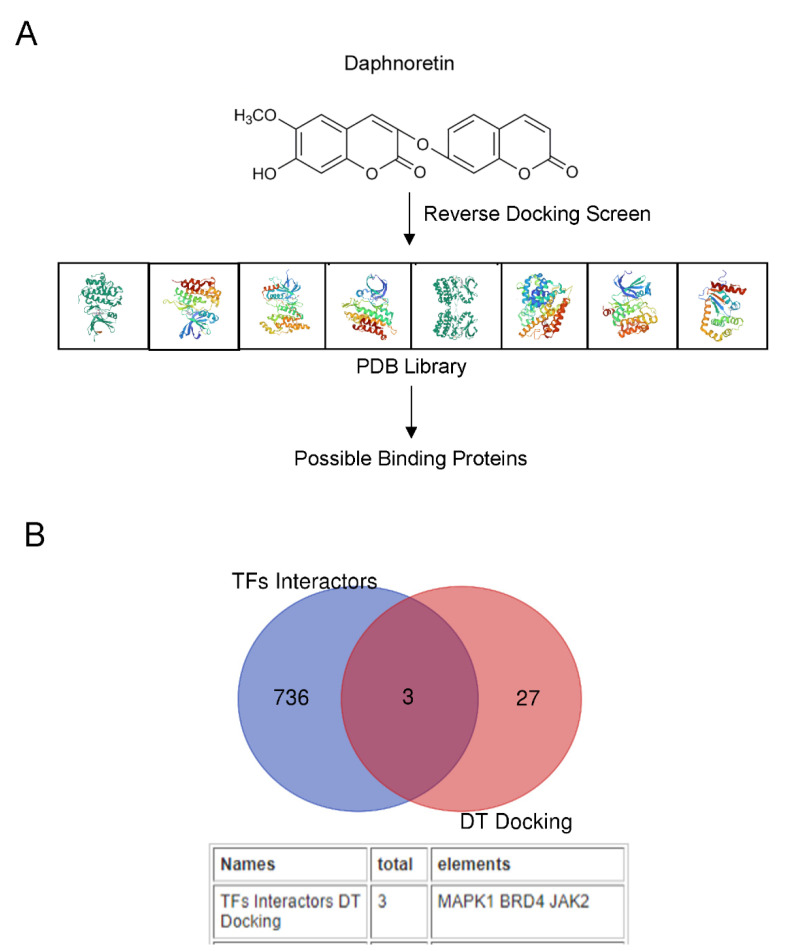
Prediction of DT possible protein targets. Scheme of DT reverse docking screen flow (**A**). Venn diagram showing protein overlap between predicted DT reverse docking proteins and predicted DT-activated TF binding proteins (**B**).

The therapeutic functions of a small-molecule drug are generally conducted by binding to cavities of proteins to influence their biological activities. Thus, the ligand-protein inverse docking approach, which utilizes 3D structural information of both the compound and the target proteins to predict their binding, can be applied to search for possible protein targets of a small molecule. Here we applied discovery studio software to further predict potential protein targets for DT (Figure 4A).

Using discovery studio software, we conducted an inverse virtual screening study for protein targets (734 crystal structures belonging to 256 proteins could be predicted for reverse docking with daphnoretin) and found that 30 proteins may directly bind with DT (Normalized Fit Score > 0.6, Table S3).

**Figure 5:**
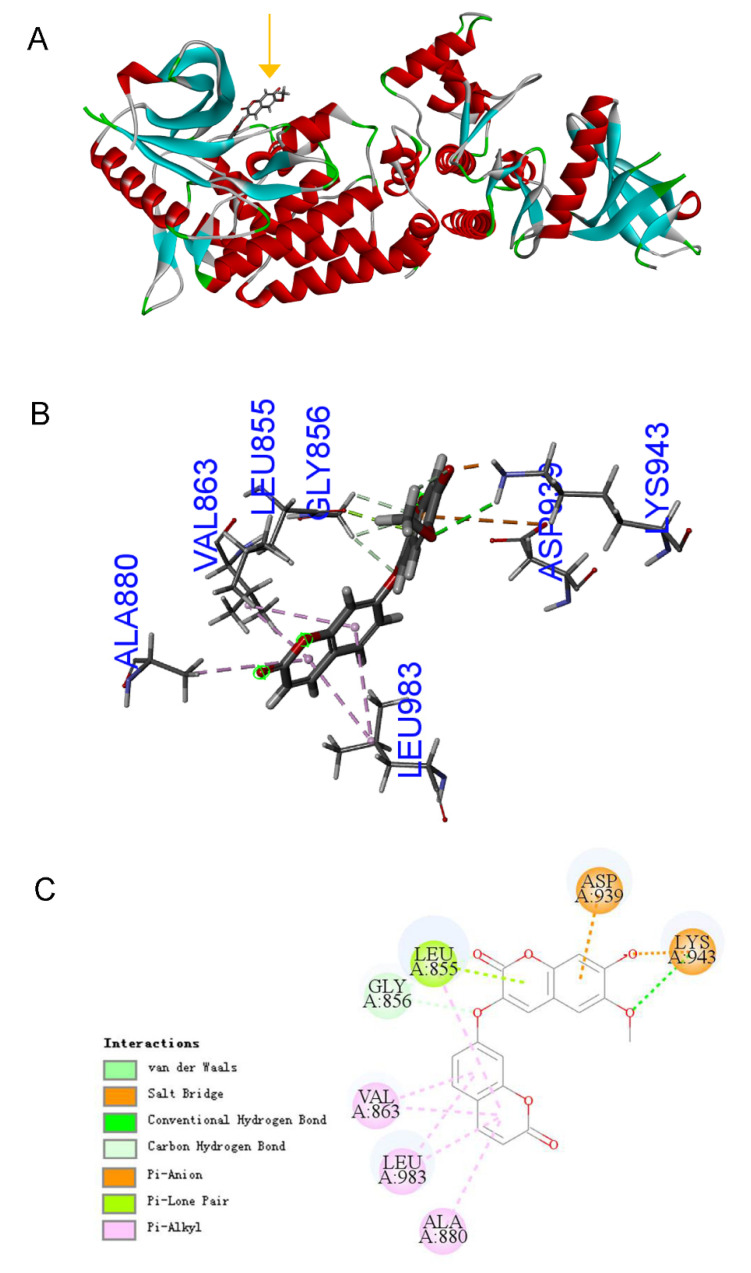
Molecular docking assay between DT and JAK2 (AA835–1132). A Protein surface front view of the docked DT in the JAK2 (PDB 2B7A) (**A**). The predicted position of the direct binding of DT and JAK2 (**B**). 2D representation of residues involved in DT binding with JAK2 (**C**).

Because we had predicted that RELA and STAT3 are the transcription factors most likely to be activated by DT, then DT should bind directly with the two TFs, or the two TFs binding proteins, to transduce the signals to TFs. We collected all 742 proteins that direct bind to RELA and STAT3 using the BioGIRD website and intersect with the predicted 30 DT docking proteins. We found that three proteins, MAPK1, BRD4 and JAK2, may directly bind to DT and activate these two TFs (Figure 4B). It has been reported that the activity of MAPK1 and BRD4 correlates with the maturation of OPCs [16,17]. The activation of the JAK2/STAT3 signaling pathway is related to the differentiation of embryonic stem cells into OLs [18]. Since it has been shown that DT mainly promotes the differentiation of NSCs into OPCs, we mainly focused on the relationship between DT and JAK2 in the follow-up study. JAK2 belongs to intracellular protein tyrosine kinases (PTKs). The kinase domain of human JAK2 (residues 835–1132) exists in either a catalytically inactive state or catalytically active state. A conformational switch repositions the highly conserved Asp-Phe-Gly motif (residues 994–996 in JAK2) in the proximity of the active site, allowing a shift in the position of the activation loop (Figure S2). These conformational states are largely governed by the phosphorylation of tandem tyrosine residues within the activation loop that results in the expulsion of the activation loop from the active site. Through molecular docking software, we found that DT fits into the hinge pocket area (AA855–943) next to the JAK2 PTK domain (AA945–1130). Its 3D structure and simplified docking diagram are shown in (Figure 5A–C), and the detailed parameters of the docking between DT and JAK2 amino acid residues are shown in Table S5. We speculate that the binding of DT to this position may promote the expulsion of the activation loop. This leads to JAK2 activation. Because it promotes the expulsion of the activation loop, activated JAK2 may more easily capture tyrosine substrates and ATP, and promote substrate tyrosine phosphorylation.

### 2.5. Wet-Lab Experiments Prove That DT Is a JAK2 Agonist

**Figure 6:**
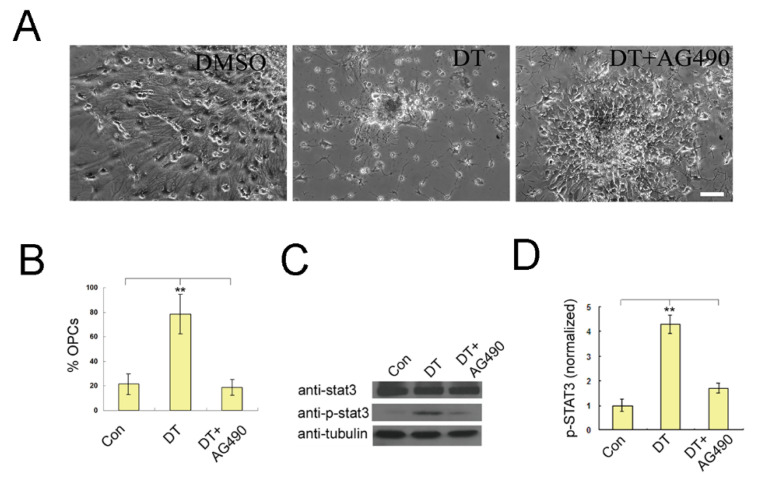
JAK2/STATA3 signaling is involved in DT-induced NSC differentiation into OPCs. Typical cell morphology of NSCs treated with DMSO, DT and DT+AG490 under a phase contrast microscope after 5 days of culture, Scale bar = 50 μm (**A**). JAK2 inhibitor AG490 treatment completely reversed the DT-induced OPC differentiation, indicating the involvement of the JAK2/STAT3 signaling, **, *p* < 0.01 by Student’s *t*-test (**A**,**B**). Western blot revealed that DT significantly stimulated the phosphorylation of STAT3 and increased the expression of olig2, while AG490 inhibited these effects, **, *p* < 0.01 by Student’s *t*-test (**C**,**D**).

**Figure 7:**
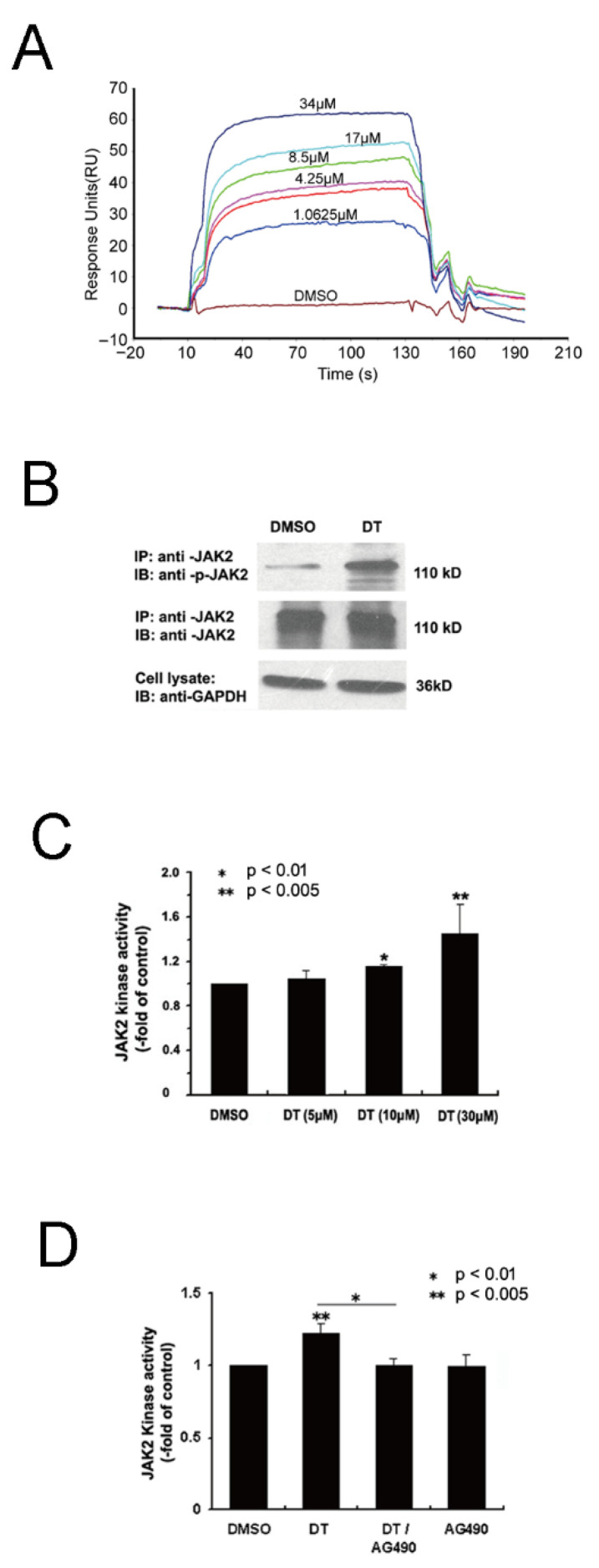
JAK2 as the protein target of DT. SPR analyses of JAK2 binding to DT. Normalized sensor grams using the JAK2 protein as the ligand and the DT as the analyte are shown. The concentrations of DT were 0, 1.0625, 2.125, 4.25, 8.5, 17, and 34 μM. The raw data (colored lines) were fitted to a 1:1 binding model (black lines) (**A**). NSC cultures pretreated with DT or DMSO (control) for 24 h were immunoprecipitated (IP) with anti-JAK2, and then immunoblotted (IB) with anti-JAK2 or anti-phospho-JAK2. An equal proportion of cell lysate was also set to detect GAPDH for input control. The representative image shows an increase in JAK2 phosphorylation in DT-treated cells in contrast to control (**B**). In vitro JAK2 kinase assay shows DT from 5 μM to 30 μM has a concentration-dependent effect on JAK2 activity. Data shown are the mean ± S.D. from three independent experiments. *, *p* < 0.01, **, *p* < 0.005 by Student’s *t* test (**C**). In vitro JAK2 kinase assay by colorimetric ELISA shows JAK2 kinase pre-incubated with DT (17 μM), DMSO, AG490 (200 nM) or DT (17 μM) plus AG490 (200 nM) has different catalytic abilities with its substrate. JAK2 kinase activities are normalized with an absorbance at 450 nm to that of the controls. Data shown are the mean ± S.D from three independent experiments. *, *p* < 0.01, **, *p* < 0.005 by Student’s *t*-test (**D**).

We hypothesized that DT may bind directly with JAK2 and activate the JAK2/STAT3 signal pathway to promote differentiation of OPCs from NSCs. To test this hypothesis, AG490, a specific inhibitor of the JAK2/STAT3 signal pathway, was added in NSC cultures combined with DT treatment. As shown in Figure 6A,B, 200 nM AG490 completely reversed DT-induced differentiation, as most of these cells displayed a large and flat morphology. We further examined the phosphorylation of STAT3 by Western blot. As shown in Figure 6C,D, DT significantly stimulated the phosphorylation of STAT3, while AG490 treatment prominently attenuated this effect. These data indicate that the activation of the JAK2/STAT3 signal pathway is essential for DT-initiated OPC differentiation. We also measured the binding kinetics of DT to purified JAK2 (Figure S2) using Surface Plasmon Resonance (SPR, Biacore). The experiments used recombinant human JAK2 (835–1132AA). As shown in Figure 7A, DT binds to JAK2 with a fast-on (16.48 s) and fast-off (12.75 s) rate and a measured K_d_ of 5.28 μM (Figure S3).

Using cell biochemistry and an in vitro kinase assay, respectively, we further investigated whether DT could regulate JAK2 activity. Primary cultured NSCs were treated with DT or an equal amount of DMSO for control. After 24 h-treatment, cultures were lysed for Western blot analysis. As shown in Figure 7B, in comparison to GADPH, there was little variance in JAK2 expression between DMSO and DT-treated cells; however, the phosphorylated JAK2 between the two groups was significantly different. Therefore, it can be concluded that DT improved JAK2/STAT3 signaling activity in cultured NSCs. To determine if DT has a direct effect on JAK2 kinase activity we conducted in vitro kinase assays in which JAK2 kinase was incubated with DT (17 μM), DMSO as control, and AG490 (200 nM) with or without DT (17 μM). Activity of substrate catalyzation was assessed by colorimetric ELISA assay. The absorbance at 450 nm measured using a microtiter plate reader was first normalized to that of the control then presented as fold of the control. As shown in Figure 7C,D, DT improved the JAK2 kinase activity significantly compared to the control treatment, (n = 3, *p* < 0.005), while AG490 interrupted its effect to some extent (n = 3, *p* < 0.01). Furthermore, our results also indicate that DT effects on JAK2 kinase activity depend on its concentration. The results support the hypothesis that DT can directly activate JAK2 kinase. In all, we now propose that DT is an agonist of JAK2 while it promotes NSC differentiation into OPCs.

## 3. Discussion

DT belongs to the coumarins group, also known as benzopyrones, which are present in remarkable amounts in plants. Coumarins have attracted much interest for a long time due to their multiple biological properties, such as anticancer effects and regulating immune activities [19,20]. In the present study, we demonstrated, to our knowledge for the first time, that DT can affect NSC differentiation.

It has always been a problem to find effective targets of TCM monomers. With the development of computer hardware and software systems, small molecule reverse docking has become an effective method for predicting TCM monomer targets, but for a single TCM monomer small molecule reverse docking software still gives dozens or even hundreds of possibilities with lots of false results. It is impossible to verify the predicted results through in vitro protein and small molecule binding experiments. In our study, we analyzed high-throughput sequencing results using bioinformatics methods, and enriched the transcription factors that may be activated or repressed according to changes in gene expression. By analyzing the known binding proteins of transcription factors and performing intersection analysis with the TCM monomer binding proteins predicted by small molecule reverse docking software, the effective targets of TCM monomers can be quickly and accurately located. We found DT may bind directly to the JAK2 protein.

Differentiation of NSCs into OLs during embryogenesis is the result of a complex interaction between cell-extrinsic signals and cell-intrinsic determinants. It has been reported that a recombinant protein, IL-6, fused to its soluble receptor, a potent activator of the gp130 receptor, can activate the JAK2/STAT3 signal pathway and strongly stimulate the differentiation of OLs if added to ES cell-derived neural precursors [18]. In the present study, when tyrphostin AG490, a specific JAK2 inhibitor, was applied, DT-induced OPC differentiation from NSCs was blocked, suggesting the involvement of the JAK2/STAT3 signaling pathway. Consistent with this observation, we found that AG490 treatment significantly attenuated DT-induced phosphorylation of STAT3. Therefore, JAK2/STAT3 signaling is crucial for DT-induced OPC differentiation from NSCs.

Next, we used SRP experiments and in vitro kinase activity assays to verify that DT can directly bind to JAK2 and promote JAK2 auto-phosphorylation and activate the JAK2/STAT3 signal transduction. Our results suggested that DT may be an agonist of JAK2. Our small molecule reverse docking computer simulation results showed that DT would fall into the hinge pocket area (AA855–943) next to the JAK2 PTK domain (AA945–1130). We speculate that the binding of DT to this position may promote the expulsion of the activation loop. This leads to JAK2 activation. Because it promotes the expulsion of the activation loop, activated JAK2 may more easily capture tyrosine substrates and ATP and promote substrate tyrosine phosphorylation. Therefore, the JAK2 inhibitor AG490, which is a tyrosine substrate analogue, can also easily access the substrate binding site. Therefore, it was shown that the lower concentration of AG490 in the in vitro activity test can inhibit the JAK2 activity caused by DT, but AG490 alone does not inhibit JAK2 (Figure 7D). Since JAK2 mutations constitutively activate downstream signaling and are drivers of myeloproliferative neoplasm [21], the discovery of specific inhibitors of the JAK2 protein has become a research hotspot for the treatment of diseases. However, no specific agonist of JAK2 has been found so far. The DT we found can specifically bind to JAK2 and promote the self-phosphorylation of the JAK2 protein and activate the JAK2/STAT3 signal transduction pathway (Figure 4). This not only provides an effective research tool for studying the mechanism of related signal transduction pathways in physiological and pathological conditions, but also may provide new therapeutic approaches for the treatment of some diseases related to JAK2/STAT3 signal pathway inhibition. As an example, IL-22 induced STAT3 phosphorylation in Lgr5 (+) ISCs after intestinal injury, and STAT3 was crucial for IL-22-mediated intestinal epithelium regeneration [22]. STAT3 is also strongly associated with liver injury and regeneration, and a STAT3 inhibitor strongly inhibited liver regeneration in hepatectomy mice [23]. Therefore, DT, as an agonist of JAK2, may play an important role in the regeneration of digestive tract epithelium, liver and the hematopoietic system.

## 4. Materials and Methods

### 4.1. NSC Culture

All procedures regarding animal care and use followed recommended NIH guidelines and were approved by the Animal Experimentation Ethics Committee of Shanghai University. Rat NSC isolation and culture were performed as described by Weiss et al. [24]. Briefly, the cortex of 1 to 2-day-old newborn SD rats were removed and placed into sterile chilled Hank’s Balanced Salt Solution. After the meninges were carefully removed, tissues were cut into tiny pieces and gently triturated with a Pasteur pipette at least 10 to 15 times. The suspended tissue was centrifuged at 500× *g* for 5 min and the pellets were re-suspended with proliferation medium (DMEM/F12 nutrient (1:1) with additional 2% B27 (Invitrogen Corporation, Carlsbad, CA, USA) supplement and 20 ng/mL bFGF (PeproTech, Rocky Hill, NJ, USA). The cells were suspended at a density of 50 cells /mL in non-coated 75 cm^2^ flasks. Every 4 days, bFGF was added along with a partial medium change. After 7 days in vitro, cells formed floating neurospheres. To subculture NSCs, the neurospheres were centrifuged at 600× *g* for 5 min when the diameters of neurospheres reached a size of approximately 100–200 μm. They were then re-suspended in fresh medium and mechanically dissociated into single cells. Single cells were seeded into a proliferation medium at a density of 10 cells/mL per flask. This procedure produced a second generation of neurospheres, and additional generations of NSCs were produced using this same procedure.

### 4.2. Traditional Chinese Medicine Monomers Library

The traditional Chinese medicine monomers library was purchased from Sunny Biotech. Co., Ltd. Shanghai, China. The monomers in the library are listed in Table S1. The company stated that the purity of the monomers was at least 98% based on HPLC, UV, MS, 1HNMR and 13C NMR. Chemical structures were identified based on their UV, MS, 1H NMR and 13C NMR data and by comparison of their spectral data with those reported previously. After we identified the compound DT, we purchased it from Sigma-Aldrich (≥98.0% (HPLC), CAS: 2034-69-7).

### 4.3. Compound Screens

Neurospheres were plated on poly-D-lysine-coated 35 mm dishes at a density of 10–20 spheres per dish in 1.5 mL medium. One hour after plating, compounds were added at a final concentration of 20 μM unless otherwise indicated. After 5 days, cells were examined with phase-contrast image or immunocytochemistry.

### 4.4. Immunofluorescence

The identification of different kinds of cells was performed using immunocytochemistry. Cells on dishes were fixed with 4% paraformaldehyde at room temperature for 20 min and washed three times in succession with 0.01 M PBS for 5 min each time. Cells were then treated with 0.3% Triton 100 containing 10% normal goat serum at room temperature for 30 min. Cells were then incubated with primary antibodies at 4 °C for 12–16 h and washed three times with 0.01 M phosphate buffered saline (PBS) for 5 min each time. Fluorescence-conjugated secondary antibodies were added to the cells, and the cells were incubated at 37 °C for 40 min. After three 5 min PBS washes, Hoechst 33258 nuclear stain was added at room temperature for 10 min, followed by two more 5 min PBS rinses. The primary antibodies included anti-GFAP (1:200; rabbit; Promega), anti-NG2 (1:200; Mouse; Chemicon), anti-O4 (1:100; Mouse; Chemicon), and anti-RIP (1:100; Mouse; Chemicon). The secondary antibodies were FITC-conjugated antibodies to rabbit IgG (1:200) and TRITIC-conjugated antibodies to mouse IgG (1:200). Immunostained preparations were examined with an inverted motorized microscope (Nikon TE2000-U) equipped for phase contrast and fluorescence. To determine the number of cells expressing a particular antigen, 100 fields per sample were examined and totaled. Results are given as mean ± S.D. of data for six samples from three independent experiments. All data were analyzed using Student’s *t* test or one-way ANOVA, and statistics were performed using GraphPad Prism 5 statistics software.

### 4.5. High-Throughput RNA Sequencing

Neurospheres were seeded onto a poly-L-lysine-coated 75 mm^2^ flask at a density of 1 × 10^4^ spheres/flask. Then, DT (34 µM) was added in the culture medium (neurobasal+B27). An equal volume of DMSO was added as a control. After culture for 5 days. total RNA was extracted from the samples with TRIzol^®^ Reagent (Invitrogen) according to the manufacturer’s instructions. Total RNA concentrations in the sample tissues and the quality of each sample were then assessed with a NanoDrop2000. Specifically, OD260/OD280 ratios between 1.8 and 2.2 were deemed acceptable, while OD260/OD230 ratios of greater than 1.8 were deemed acceptable. RNA integrity and DNA contamination were assessed using electrophoresis on a denaturing agarose gel. RNA-seq library preparation carried out using the TruSeq™ Stranded Total RNA Library Prep Kit for Illumina.

### 4.6. Bioinformatics Analysis and Target Prediction

We used the website (https://maayanlab.cloud/chea3/ and https://metascape.org/gp/index.html#/main/step1, accessed on 5 November 2021) to analyze the differentially expressed genes in our transcriptome sequencing results and target the transcription factors that may be activated by DT.

### 4.7. Inverse Virtual Screening

The X-ray crystal structures of proteins were obtained from the Protein Data Bank (PDB) (http://www.rcsb.org/pdb, accessed on 4 May 2021). The heteroatoms (i.e., non-receptor atoms such as water and ions) and conformers present in the crystal structure were removed, and the invalid residues were fixed for preparing the protein for docking. We imported the DT mol format file into Discovery studio software, and used the software to simulate molecular-target protein docking, keeping the default settings of the parameters. The software automatically matched DT with the protein PDB model that was imported in its database, and ranked it according to the normalized fit value.

### 4.8. Immunoprecipitation and Immunoblotting

Harvested cells were washed twice with cold phosphate-buffered saline and solubilized with ice-cold lysis buffer (50 mM Tris-HCl, pH 8.0, 1 mM EDTA, 150 mM NaCl, 0.5% Na-deoxycholate, 0.02% Na-azide, 1 mM NaF, 1 mM Na-vanadate, 1 mM phenylmethylsulfonyl fluoride, 1% Nonidet P-40, 1 mM dithiothreitol, 0.1% SDS, 2 μg/mL pepstatin, 2 μg/mL leupeptin and 2 μg/mL aprotinin). Lysates were clarified by centrifugation at 11,200× *g* for 10 min at 4 °C. The protein concentration of the supernatants was determined by the Bradford method. For immunoblotting, 10 μL of supernatant was subjected to SDS-PAGE, immunoblotted, and visualized with enhanced chemiluminescence (ECL, Pierce). For immunoprecipitation, 300–500 μL of supernatant was incubated with 5 μL of the corresponding antibody for 3 h at 4 °C. Protein G-agarose beads (Roche) were then added for 3 h. Immunoprecipitated samples were then washed three times with lysis buffer, boiled 3–5 min in the sample-loading buffer, and then subjected to Western blotting analysis. The antibodies are listed as follow: Jak2 #74987,#3230 CST, pJak2 #3771 CST, STAT3 #4904 CST, pSTAT3 #9131 CST, αTubulinsc-53646, GAPDH ab8245 Abcam.

### 4.9. Determination of Binding Affinity of DT to JAK2 Using Splasmon Resonance Techniques

Surface plasmon resonance (SPR) experiments were carried out with a BIAcore 8k (GE Healthcare, Uppsala, Sweden) using immobilized purified JAK2 protein on a BIAcore CM5 sensor chip. The immobilization of His-JAK2 was performed at 25 °C, and HBS-EP+ (10 mM HEPES, 150 mM NaCl, 3 mM EDTA, and 0.05% P20, pH 7.4) was used as the running buffer. Briefly, both flow cells of channel 2 of the sensor chip were activated by freshly mixed 50 mmol/L N-hydroxysuccinimide and 200 mmol/L 1-ethyl-3-(3-dimethylaminopropyl) carbodiimide hydrochloride for 300 s (10 μL/min). Then, 2 μg/mL His-JAK2 diluted in 10 mmol/L NaAC (pH 4.0) was injected into the flow cell 2 to reach a level of approximately 100 units of response, while flow cell 1 was set as the blank. After the amine coupling reaction, the remaining active coupling sites were blocked with a 300-s injection of 1 mol/L ethanolamine hydrochloride. The measurement was performed at 25 °C, and HBS-EP+ was used as the running buffer. An injection of the tested analyte and surface regeneration of the sensor chip was included in each running cycle. Serially diluted DT (DT 0, 1.0625, 2.125, 4.25, 8.5, 17, and 34 μM) were injected sequentially into both cells of channel 2 (30 μL/min) with an association time of 180 s, and buffer flow was maintained for 600 s for dissociation. For regeneration, a duplicate 30-s injection of 10 mM glycine-HCl (pH 1.5) was used. KD values were calculated with BIAcore 8k evaluation software 1.0 with a 1:1 binding model.

### 4.10. JAK2 In Vitro Kinase Activity Assay

Kinase assays were conducted with recombinant Jak2 and Jak2 substrate using HTScan^®^ JAK2 Kinase Assay Kit (#7752, Cell Sgnaling Tec, Danvers, IL, USA). The DT effect on kinase activity was measured according to the manufacturer’s instruction.

### 4.11. Statistics

All data were subject to statistical analysis using one-way analysis of variance (ANOVA) supplemented with Student’s *t* tests (GraphPad Prism 5 statistics software). All the data are shown as mean ± SEM from at least three experiments (*p* < 0.05 is considered to represent a significant difference).

## 5. Conclusions

In summary, we developed a bioinformatics analysis method which can quickly and accurately locate the effective targets of TCM monomers. In the present study, we identified a small molecule, DT, from traditional Chinese herbs, which could significantly promote cultured NSCs to differentiate into OPCs. DT could bind directly with JAK2 and act as an agonist to activate JAK2. The activation of the JAK2/STAT3 signal pathway was demonstrated to be crucial for DT-induced OPC development from NSCs. Therefore, these findings may expand the therapeutic potential of NSCs for demyelinating diseases and traumas.

## Data Availability

The data and materials used in the current study are available from the corresponding author upon reasonable request.

## Supplementary Materials

The following supporting information can be downloaded at: https://www.mdpi.com/article/10.3390/molecules27186105/s1, Figure S1: NSCs treated with or without DT (34 μM) for 5 days, then the derived cell stained with oligodendrecytes pre-mature marker(O4), and mature oligodendrecytes marker (RIP). Figure S2: His-JAK2 kinase domain expression and purification. Figure S3: DT and JAK2 binding curve and Kd value fitted according to DT concentration. Table S1: The monomers in the library are list for screen. Table S2: List of differentially expressed genes for the first transcriptome sequencing. Table S3: List of differentially expressed genes for the second transcriptome sequencing. Table S4: A list of potential protein targets that binding with DT predicted by Discovery studio software and arranged in descending order according normalized Fit Score. Table S5: The interactions, distances, and nature of hydrophobic or hydrophilic amino acids in Figure 5C.

## Sample Availability

Samples of the compounds used in the current study are available from the authors.

## Abbreviations

ESCs: Embryonic stem cells; MS: Multiple Sclerosis; OPCs: Oligodendrocyte Precursor Cells; NSCs: Neural Stem Cells; SPR: Surface Plasmon Resonance; DIV: Days In Vitro; DT: Daphnoretin; DMEM: Dulbecco’s Modified Eagle Medium; FBS: Fatal Bovine Serun.
