# Supplementary material for: Comprehensive Bioinformatics Analysis Combined with Wet-Lab Experiments to Find Target Proteins of Chinese Medicine Monomer
Source: Molecules. 2022 Sep 19;27(18):6105. doi: 10.3390/molecules27186105 (PMC9504604; doi:10.3390/molecules27186105)
Supplement: Supplementary file 1 [file molecules-27-06105-s001.zip › Supplementary materials.pdf]

molecules

Article

Comprehensive Bioinformatics Analysis Combined With Wet-Lab Experiments To Find Target Proteins Of Chinese Medicine Monomer

Xiao-Hui Xu[1#], Yun-Yi Zhu[2#], Chang-ling Yue[2#], Qian-wen Yang[2#] and Zhao-Huan Zhang[3*]

Supplementary methods

Detailed methods and preliminary results for screening of traditional Chinese medicine monomer library to promote the directed differentiation of NSCs.

Neural stem cell spheres were differentiated on a 12-well plate coated with PDL, and a compound at a concentration of 20uM was added for differentiation screening experiment. Because neural stem cells can only differentiate into neurons, astrocytes and oligodendrocytes, and the morphological differences of these three cells are particularly obvious when observed under a phase contrast microscope, so they are very suitable for manual screening. In fact, we originally wanted to find compounds that promote the differentiation of neural stem cells into neurons, but from the library molecules we screened, we did not find any compounds that obviously promote neuronal differentiation. But what shocked us was that we discovered a compound that was later determined to be that DT could significantly promote the differentiation of NSCs into small cells with 2-6 short branches (Fig 3a). After immunofluorescence identification, we thought it should be the precursor cell of oligodendrocytes, and DT can maintain it in the state of precursor cells for a long time in the differentiation medium(Fig 1a-f). We have not observed obvious cell division, which means that DT may not to promote OPCs proliferation in the differentiation medium(neurobasl+2%B27).

In vitro analysis to derive the DT and JAK2 binding curve and Kd value

The kinase domain of human JAK2 (residues 835-1132 [PubMed NM004972]) was cloned into pFastBac, which allows the protein to be expressed fused to a His6 tag in (Sf9) insect cells. The cells harvested 48 hours after infection. Cells were resuspended into a buffer consisting of 20 mM Tris HCl, pH 8.5, 250 mM NaCl, 0.5% thesit, 5% glycerol, and 1 mM DTT supplemented with complete protease inhibitors mixture (Roche Diagnostics, Mannheim, Germany), lysed by sonication, and centrifuged at 45 000g for 1 hour. The supernatant was filtered and load to affinity column containing Talon IMAC beads (Clontech) and eluted with a gradient of 5–500 mM imidazole. After extensive washes, the fusion protein was eluted, and fractions containing JAK2 were collected. Then the protein concentration and removing the Imidazole with Centrifugal Filter Devices (Ultra-15(10kD),Millipore).

Supplementary Tables

Supplementary Table S1. The monomers in the library are list for screen.
Supplementary Table S2. List of differentially expressed genes for the first transcriptome sequencing.
Supplementary Table S3. List of differentially expressed genes for the second transcriptome sequencing.
Supplementary Table S4. A list of potential protein targets that binding with DT predicted by Discovery studio software and arranged in descending order according normalized Fit Score.
Supplementary Table S5. The interactions, distances, and nature of hydrophobic or hydrophilic amino acids in Fig 5C.

Supplementary figures

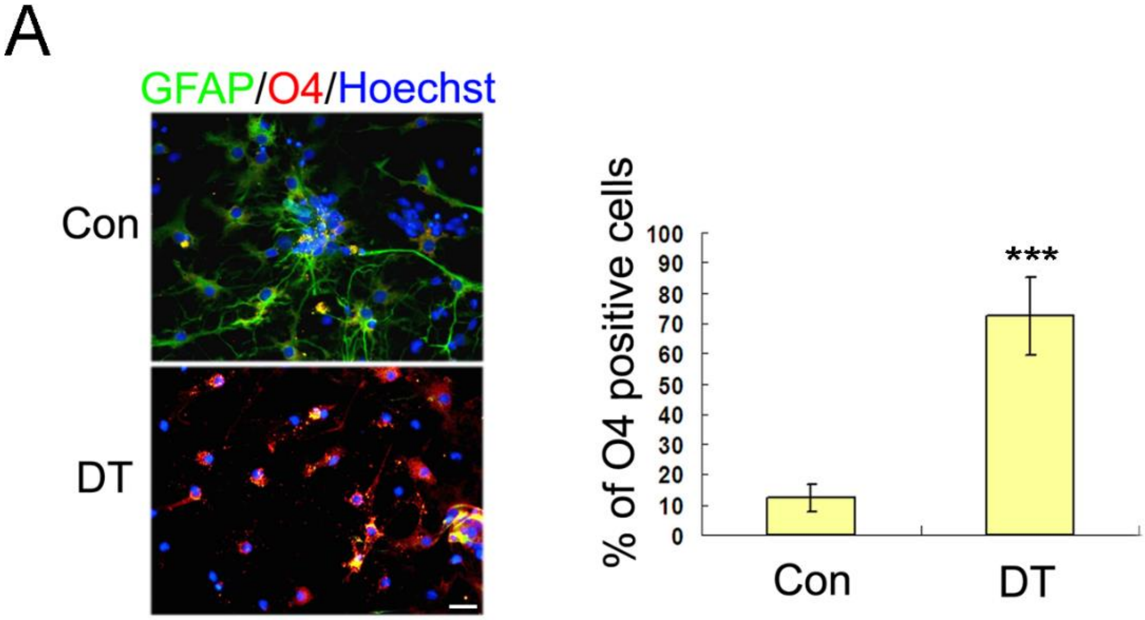

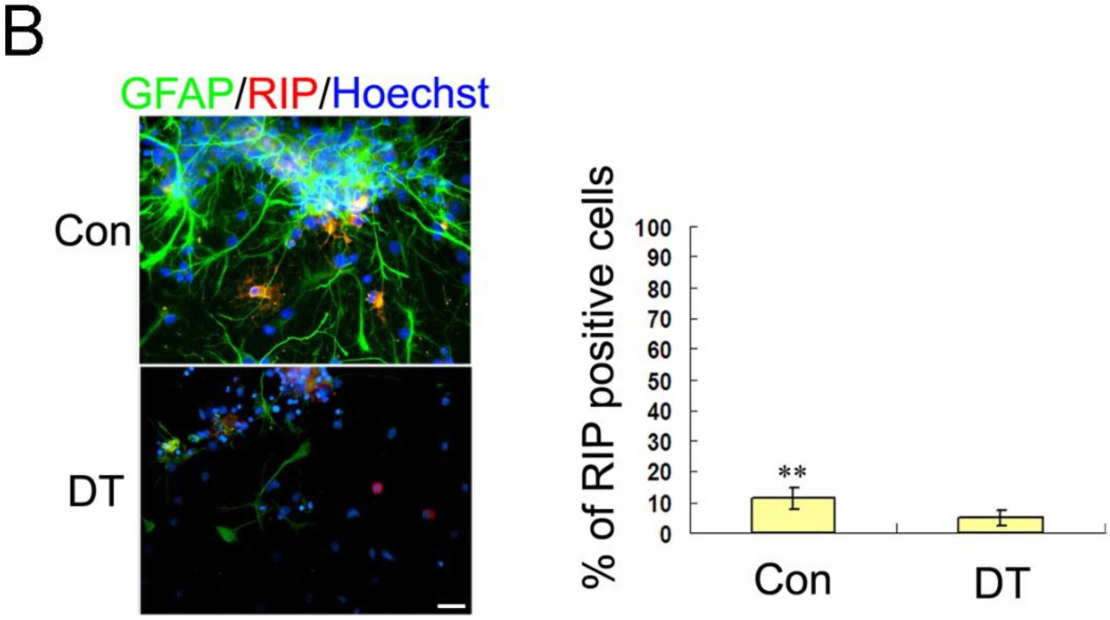

Figure S1. NSCs treated with or without DT（34μM）for 5 days， then the derived cell stained with oligodendrecytes pre-mature marker(O4), and mature oligodendrecytes marker (RIP). Most of these derived cells were O4 positive (c,d), but not RIP positive (e, f), indicating the characterization of premature oligodendrocytes. Data are presented as means ± S.D. **P < 0.01, ***P < 0.0 = 1 vs controls. Scale bar = 25 μm.

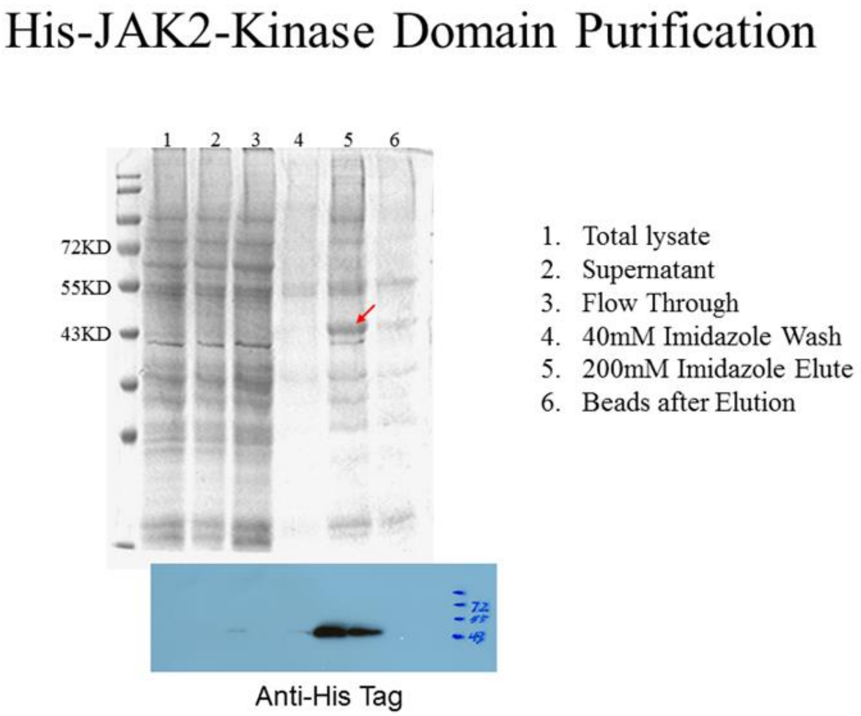

Figure S2. His-JAK2 kinase domain expression and purification.

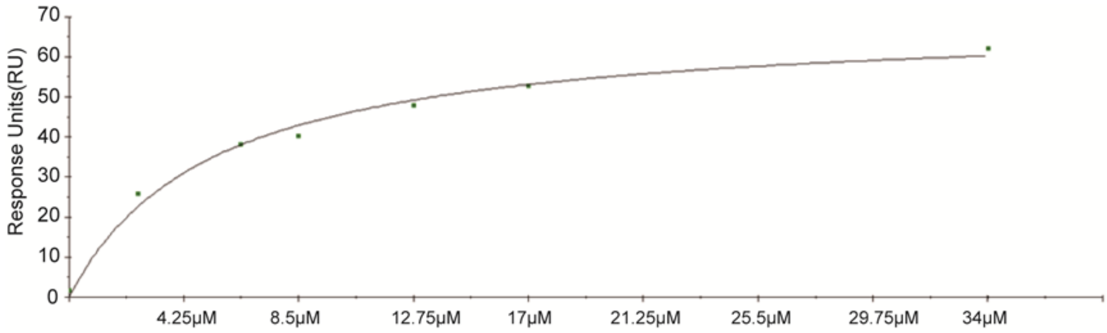

Figure S3. DT and JAK2 binding curve and Kd value fitted according to DT concentration.
